# Quantifying the magnitude of hazardous incidents among laboratory staff in Kenya; preliminary results of a national health care workers survey, 2014-2015

**DOI:** 10.1186/2047-2994-4-S1-P97

**Published:** 2015-06-16

**Authors:** BK Burmen, J Osoga

**Affiliations:** 1 HIV and TB Implementation Science and Services, Kenya Medical Research Institute/Centers for Disease Control Research and Public Health Collaboration, Kisumu, Kenya

## Introduction

Occupational health surveillance data are vital to effective intervention. Limited information is available on the magnitude of occupational injuries among laboratory personnel in Kenya.

## Objectives

We set out to quantify the prevalence of hazardous injuries among laboratory personnel in Kenya.

## Methods

As part of the Kenya’s premier national public health laboratory’s training on bio-safety and bio-security, laboratory personnel were invited to take part in a survey on occupational hazards and the safety climate at their workstations. Descriptive statistics were used to summarize types of hazardous incidents experienced by laboratory personnel. Logistic regression was used to describe factors associated with reporting a hazardous injury.

## Results

One hundred and sixteen laboratory personnel drawn from 108 heath facilities participated. Majority were drawn from public health facilities (90%); the others were from private health facilities (8%) and faith based organizations (2%). Twenty-five (22%) were from facilities that had reporting mechanisms for occupational exposures. The median duration of service was 4 years (Range 0.2-33.0) and 18 (16%) had ever been trained on bio-safety. Eighty-nine (77%) personnel experienced by 127 incidents, these were: spills (46), sharps injuries (38), hazardous gas inhalation (19), subcutaneous chemical exposures (17), falls (6) and hazardous agents ingestion (1). Incidents occurred during spillage (44%), laboratory procedures (35%), waste handling (28%), surface-contamination (22%), maintaining equipment (9%), device use (7%),while others were due to inappropriate dressing (8%), food stuff in work area (4%), fires (2%) and heavy lifting (1%). At the time of incident, PPE donned by the majority were gloves (87%) and lab coats (82%). Only 63% (56) reported their incidents; sharp injuries were more likely to have been reported (OR 3.1 95% CI 1.4-7.0, p < 0.05).

## Conclusion

Due to the magnitude of occupational hazards, an integrated information and incident management system should be implemented to routinely document occupational hazards.

## Disclosure of interest

None declared.

